# Fatty acid-binding protein-4 (FABP4) and matrix metalloproteinase-9 (MMP9) as predictive values for nonalcoholic steatohepatitis (NASH)

**DOI:** 10.1186/s12944-022-01764-1

**Published:** 2023-01-06

**Authors:** Jonas Wagner, Yogesh Kumar, Anne Lautenbach, Philipp von Kroge, Stefan Wolter, Oliver Mann, Jakob Izbicki, Nicola Gagliani, Anna Duprée

**Affiliations:** 1 Department of General-, Visceral- and Thoracic Surgery, University Medical Center Hamburg-Eppendorf, Martinistr. 52, 20246 Hamburg, Germany; 2 Department of Medicine, University Medical Center Hamburg-Eppendorf, Martinistr. 52, 20246 Hamburg, Germany; 3 Department of Medicine, Section of Molecular Immunology und Gastroenterology, University Medical Center Hamburg-Eppendorf, 20246 Hamburg, Germany

**Keywords:** Bariatric surgery, Liver pathology, NAFLD, NASH, Biomarker, MMP9, FABP4

## Abstract

**Background:**

Nonalcoholic fatty liver disease (NAFLD), especially nonalcoholic steatohepatitis (NASH) increases the risk for liver cirrhosis. Noninvasive tests for NAFLD/NASH exist, but they are unreliable and thus liver biopsy remains the standard for diagnosis and new noninvasive diagnostic approaches are of great interest. The aim of this study was to test whether the serum levels of fatty acid-binding protein-4 (FABP4) and matrix metalloproteinase-9 (MMP9) could be used as a diagnostic tool for NASH.

**Methods:**

Patients who underwent bariatric surgery and simultaneous liver biopsy were identified. Biopsies were assigned a NAFLD activity score (NAS). MMP9- and FABP4- Enzyme-linked Immunosorbent Assays (ELISAs) on serum samples were performed. The serum levels of FABP4/MMP9 were compared and different models to predict NASH were developed.

**Results:**

A total of 84 patients were included, 28 patients (33.3%) were diagnosed with NASH. Higher concentrations of MMP9 in NASH patients (*p* < 0.01) were detected. FABP4 concentrations were not significantly increased. A moderate correlation between the NAS and MMP9 concentrations (*r* = 0.32, *P* < 0.01) was observed. The neural network model fit best with the dataset, with an area under the curve (AUC) of 83% and an accuracy of 88%.

**Conclusion:**

Serum MMP9 levels are increased in patients with NASH and should routinely be measured in patients with obesity, but further investigations are needed to improve noninvasive NASH diagnosis.

## Background

Nonalcoholic fatty liver disease (NAFLD) is characterized by pathological hepatic lipid accumulation in the absence of alcoholic liver disease or other liver diseases [1]. It is estimated that approximately 25–45% of the worldwide population suffers from NAFLD, making NAFLD one of the most important global health challenges [2, 3]. The prevalence of NAFLD increases in patients with obesity and it is estimated that between 75 and 100 million individuals suffer from NAFLD in the United States [3].

NAFLD can be subdivided into three main categories based on histological findings. The nonalcoholic fatty liver (NAFL) category includes patients with steatosis and mild inflammation. The nonalcoholic steatohepatitis (NASH) includes patients with additional histological characteristics, such as the ballooning of hepatocytes. The last category includes patients with characteristics of both NAFL and NASH, hence termed the borderline category. Additionally, some patients show histological features of liver fibrosis, which can also be scored on a scale from absent (score of 0) to liver cirrhosis (score of 4) [1]. Subtype classification has clear implications for disease progression. While less than 4% of patients with NAFL progress to liver cirrhosis, approximately 20% of patients with NASH progress to liver cirrhosis [4].

The current standard for diagnosis remains liver biopsy [5]. However, it is not feasible to perform liver biopsy in such an immense number of patients. Although some tests already exist, none show enough diagnostic accuracy for routine clinical use [5]. Since patients with the disease are mostly asymptomatic, without accurate biomarkers, NAFLD can progress unnoticed to more advanced stages and cirrhosis [6]. Therefore, it is of great interest to develop reliable noninvasive tests.

Additionally, noninvasive markers are needed for drug development. On one side, these markers could be used to monitor disease severity. On the other side, these markers could be potential drug targets [7].

In a recent paper, Coilly et al. reported that the hepatic expression of fatty acid-binding protein-4 (*FABP4*) and matrix metalloproteinase-9 (*MMP9*) helped to accurately predict the disease progression of patients with NAFLD [8]. However, little is known about the serum concentrations of these proteins in NAFLD, and their diagnostic values in circulation remain to be determined. Fatty acid-binding proteins are lipid chaperones and orchestrate lipid trafficking. FABP4 is the isoform of FABP that is mainly expressed in adipocytes, and it has already been suggested to play a role in metabolic diseases such as type 2 diabetes (T2D) [9, 10]. MMP9 belongs to the matrix metalloproteinases, which are proteins that are involved in the degradation of the extracellular matrix [11]. MMP9 has been implicated in various malignancies [12–14], including hepatocellular carcinoma [15]. However, few studies investigated the circulating protein levels of either MMP9 or FABP4 in the context of histologically proven NAFLD [16–18].

The aim of this study was to assess the circulating levels of FABP4 and MMP9 in patients with NAFLD and associate them with disease severity. The second aim was to generate a predictive test for NASH.

## Methods

### Patient selection

This retrospective study was conducted with patients undergoing bariatric surgery in a Center of Excellence for bariatric surgery. 84 patients were selected from a biobank for whom liver biopsy and simultaneous serum samples were available. Anthropometric data, comorbidities, type of surgery and standard laboratory parameters were collected. All patients were screened before surgery by a multidisciplinary team consisting of an endocrinologist, a psychologist, a nutritionist, a physical therapist, and a surgeon. Patients were selected for surgery if they had a body mass index (BMI) ≥ 40 kg/m² or ≥ 35 kg/m² and related comorbidities in accordance with the German Guidelines of the Surgical Treatment of Obesity after discussion with an interdisciplinary obesity board. Certified bariatric surgeons performed all surgical procedures. The procedure was chosen depending on BMI, comorbidities, medications and patient request [19, 20]. In cases of suspected liver pathology, intraoperative biopsies were obtained. The biopsies were examined by an independent senior specialist in gastrointestinal pathology to diagnose NAFLD/NASH based on the NAFLD activity score (NAS) according to Kleiner et al. [1]. Simultaneously-collected serum samples were frozen and stored at -80 °C until further analysis.

The patients were grouped based on the NAS. Patients with scores above four (≥ 5) were diagnosed with NASH, patients with scores of three and four were considered borderline, and patients with scores below three were deemed healthy obese (HO) patients or patients with NAFL.

### Enzyme-linked Immunosorbent Assays (ELISAs)

ELISAs for FABP4 (AssayPro) and MMP9 (Sigma-Aldrich) serum samples from patients with available NASs were performed according to the manufacturer’s protocol. In brief, the samples were thawed and diluted according to the protocol. Samples and standards were added to the wells of a precoated ELISA plate. After washing, the respective biotinylated antibodies were added. Following recommended incubation and another washing step, streptavidin-peroxidase conjugates were added to the wells. After the final washing step, the enzyme substrate was added to the wells, the reaction was stopped, and the intensity of the color was detected on a FLOUstar Omega microplate reader (BMG Labtech).

The primary outcome was the serum levels of FABP4 and MMP9 in the respective NAFLD groups. Additionally, the relationship between the two markers and the NAS was explored. As a secondary outcome it was investigated whether the markers could predict patients with NASH (NASs > 4).

The institutional review board approved the biobank and study. All patients gave their informed consent

### Statistics

Statistical analysis was performed with the Statistical Package for Social Sciences software (SPSS; IBM, Version 24), R Version 4.1.1 (R Foundation) and GraphPad Prism (GraphPad Software, Inc., Version 6). Patient characteristics are presented overall as the mean ± standard deviation (SD) for continuous variables. For comparisons between continuous variable groups, analysis of variance (ANOVA) was performed. The χ² test was used to analyze differences between nominal data. Pearson correlation analysis was performed to detect the relationship between clinical and laboratory values, including serum FABP4/MMP9 levels and the NAS. Mathematical models were developed to predict NASs > 4 based on features selected from the Pearson correlation analysis. All patients, with NAS as the endpoint, were considered for modeling. The data were normalized using the formula [xnormalized = (x-x-min)/(xmax – xmin). The data were subsequently randomly divided into training (80%) and test datasets (20%) to perform 5-fold cross-validation. Furthermore, the model was developed using four different supervised machine learning approaches, namely support vector machine (SVM), random forest (RF), neural network (NN) and decision tree (DT) approaches [21, 22]. *P*-values < 0.05 were considered to be statistically significant. The models were evaluated based on the receiver operating characteristic area under the curve (ROC AUC).

## Results

### Characterization of the NAFLD cohort

The baseline characteristics are displayed in Table 1. In this study 84 patients were included. The average NAS was 3.2 ± 2.2. Average serum MMP9 and FABP4 levels were 9.9 ± 5.2 µg/mL and 30 ± 19.4 ng/mL, respectively. NAFLD was the most common obesity-associated comorbidity (84.5%), followed by dyslipidemia (71.4%), hypertension (60.7%), type 2 diabetes (45.2%) and obstructive sleep apnea syndrome (29.8%).

**Table 1:** Patient characteristics

n	84
**Age [years]**	41.3 ± 11.4
**Weight [kg]**	155 ± 29
**Height [cm]**	173 ± 11
**BMI [kg/m**^**2**^**]**	51.4 ± 7.8
**Women [n/%]**	58/69
**Men [n/%]**	26/31
**Type 2 diabetes [n/%]**	38/45.2
**Hypertension [n/%]**	51/60.7
**Dyslipidemia [n/%]**	60/71.4
**OSAS [n/%]**	25/29.8
**Healthy [n/%]**	13/15.5
**NAFL [n/%]**	19/22.6
**Borderline [n/%]**	24/28.6
**NASH [n/%]**	28/33.3
**NAS**	3.2 ± 2.2
**MMP9 [µg/mL]**	9.9 ± 5.2
**FABP4 [ng/mL]**	30 ± 19.4

To investigate whether MMP9 or FABP4 levels are associated with NAFLD severity, the patients were divided into three groups based on the NAS (HO/NAFLD, borderline or NASH). Twenty-eight patients (33.3%) had histologically proven NASH, and the rest were divided into the healthy liver, NAFL or borderline histology groups. Patients with advanced NAFLD stages suffered more often from dyslipidemia (*P* < 0.01) and type 2 diabetes (*P* < 0.01). The group characteristics are displayed in Table 2.

**Table 2:** Group characteristics

	HO/NAFL	Borderline	NASH	*P*-value
**n**	32	24	28	
**Age [years]**	42.8 ± 12	39.5 ± 12	41.1 ± 9.5	0.55
**Weight [kg]**	148 ± 28.5	156 ± 23.8	163 ± 32.8	0.13
**Height [cm]**	173 ± 12	173 ± 10	174 ± 12	0.99
**BMI [kg/m**^**2**^**]**	49.2 ± 8.4	51.7 ± 5.8	53.8 ± 8.2	0.07
**Women [n/%]**	22/68.75	16/66.7	20/71.4	0.93
**Men [n/%]**	10/31.25	8/33.3	8/28.6	0.93
**Type 2 diabetes [n/%]**	9/28.1	10/41.7	19/67.9	0.01
**Hypertension [n/%]**	18/56.3	12/50	21/75	0.15
**Dyslipidemia [n/%]**	14/43.8	20/83.3	26/92.9	< 0.001
**OSAS [n/%]**	10/31.3	7/29.2	8/28.6	0.78
**NAS**	0.8 ± 0.8	3.5 ± 0.5	5.6 ± 0.8	< 0.001
**MMP9 [µg/mL]**	8.1 ± 3.5	9.1 ± 3.7	12.6 ± 6.5	0.002
**FABP4 [ng/mL]**	25.2 ± 9.1	31 ± 18.5	34.6 ± 26.8	0.166

### MMP9 concentrations are increased in NASH

**Fig. 1:**
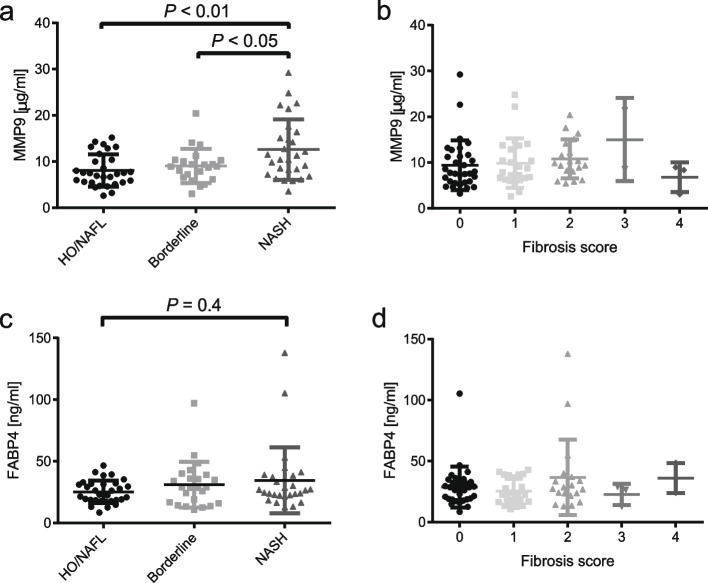
MMP9 concentrations in the NAS groups (**A**) and by fibrosis score **B**. FABP4 concentrations in the NAS groups (**C**) and by fibrosis score (**D**)

**Fig. 2:**
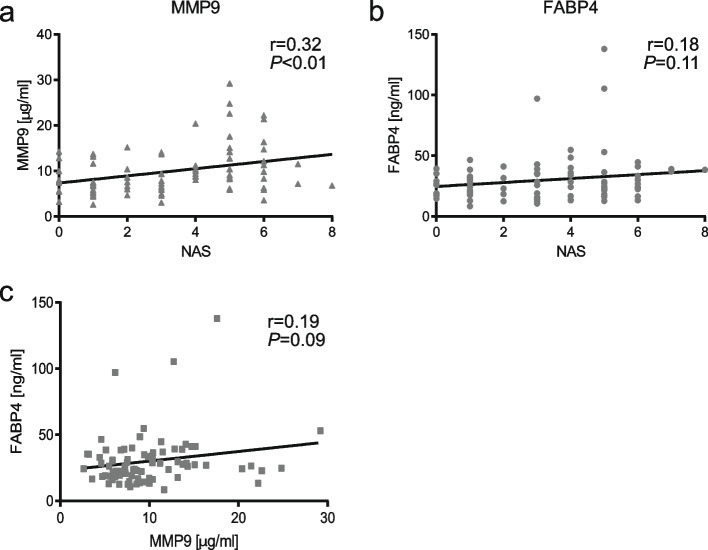
Scatter plots and Pearson correlation for MMP9 concentrations and the NAS (**A**), FABP4 concentrations and the NAS (**B**) and FABP4 and MMP9 concentrations (**C**)

Increased serum MMP9 levels in patients with NASH compared to borderline (*P* < 0.05) and NAFL/HO patients (*P* < 0.01) were observed (Fig. 1 A). No significant difference for fibrosis scores among the groups was found (Fig. 1B). MMP9 concentrations were not different between sexes in the NAFL/HO patients (female: 8.1 ± 3.1 µg/mL vs. male: 8.3 ± 5.6 µg/mL) (*P* = 0.92). Serum FABP4 levels were not elevated in patients with NASH compared to healthy controls/NAFL (Fig. 1 C/D). To further validate that MMP9 levels are associated with NAFLD severity, MMP9 levels and the NAS were correlated. MMP9 levels were indeed positively correlated with the NAS (*r* = 0.32, *P* < 0.01) (Fig. 2 A). The NAS contains three categories steatosis, ballooning and inflammation. It remained unclear which of these categories was the most important one, therefore MMP9 levels were correlated with each category. Only the inflammation score showed a significant positive correlation with MMP9 concentrations (*r* = 0.23; *P*-value = 0.049). FABP4 levels did not correlate with the NAS or MMP9 levels (Fig. 2B/C). Furthermore, FABP4 concentrations did not correlate with the inflammation, steatosis or ballooning score (*P*-values: inflammation = 0.08; steatosis = 0.19; ballooning = 0.2).

### Predictive value of MMP9

**Fig. 3:**
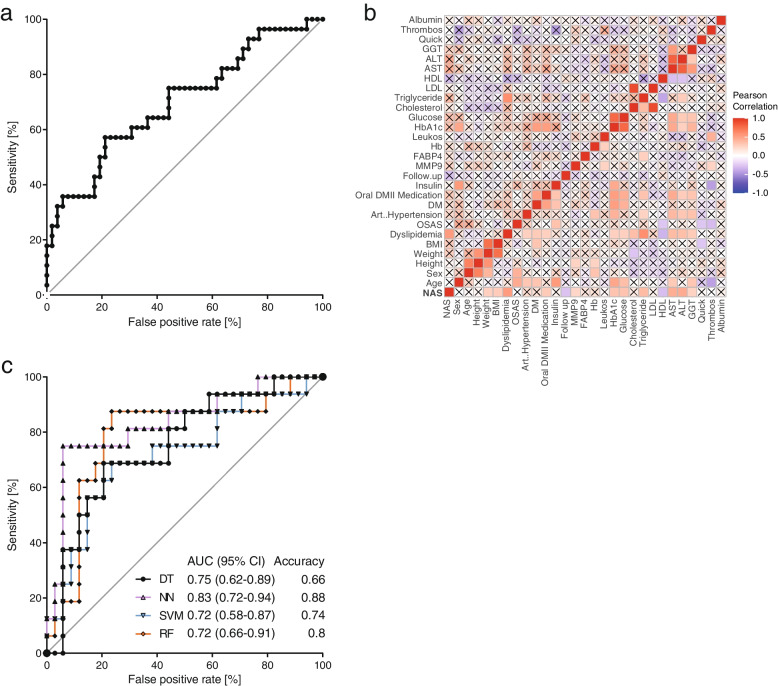
ROC curve for serum MMP9 concentrations **A**. Correlation matrix for all variables. Red indicates positive correlation, blue negative correlation. X marks non-significant correlation values **B**. ROC curve for different models. AUCs and, accuracies for each model are indicated (**C**)

To assess the predictive value of MMP9 concentrations, ROC analysis was performed and an AUC of 0.7 (95% confidence interval (CI): 0.58–0.82) was calculated (Fig. 3 A). To further improve the predictive value of MMP9 concentrations, different models were built. As the first step, additional clinical parameters were correlated with the NAS and the 12 most promising candidates were chosen (Table 3; Fig. 3B). Weight, BMI, dyslipidemia, triglyceride levels, hemoglobin A1c (HbA1c), oral diabetes medication use, glucose levels, high-density lipoprotein (HDL) levels, aspartate transaminase (AST) levels, alanine transaminase (ALT) levels, gamma-glutamyltransferase (GGT) levels and MMP9 levels were included as independent variables. Using different algorithms, the models predicted whether patients had a NAS > 4. The overall performance of these models was good, with AUCs from 0.72 (SVM) (95% CI: 0.58–0.87) to 0.83 (95% CI: 0.72–0.94) (NN) and accuracies up to 0.88 (NN) (Fig. 3 C). The sensitivity and specificity were calculated to check the performance of the models. The sensitivity and specificity reached the following values SVM (0.68, 0.79), DT (0.69, 0.65), RF (0.81, 0.79), and NN (0.75, 0.94). Additionally, this study sought to determine whether serum lipids, ALT, AST and BMI might influence MMP9 levels. Therefore, Pearson correlation for these markers was calculated, but none showed significant correlation (Table 4). Conversely, MMP9 levels were not different between patients with and without dyslipidemia (*P* = 0.29). Patients with T2D had significantly higher NAS values (2.5 ± 2.1 vs. 4.2 ± 1.9) (*P* < 0.001). MMP9 concentrations did not correlate with HbA1c concentrations (Table 4), but patients with diabetes had increased circulating MMP9 levels (8.8 ± 4.1 µg/mL vs. 11.4 ± 6.2 µg/mL) (*P* = 0.03).

**Table 3:** *P*-values for the correlation matrix. Bold values are included in the model

	*P*-value
NAS	0
Age [y]	0.99
Sex	0.83
Height [m]	0.69
**Weight [kg]**	**0.04**
**BMI [kg/m²]**	**0.03**
**Dyslipidemia**	**< 0.01**
OSAS	0.87
Hypertension	0.1
Type 2 diabetes	0.16
**Oral diabetes medication**	**0.02**
Insulin treatment	0.63
**MMP9 [µg/mL]**	**< 0.01**
FABP4 [ng/mL]	0.24
Hemoglobin [g/dL]	0.19
Leucocytes [n/L]	0.74
**HbA1c [%]**	**< 0.01**
**Glucose [mg/dL]**	**< 0.01**
Cholesterol [mg/dL]	0.36
**Triglycerides [mg/dL]**	**< 0.01**
LDL [mg/dL]	0.54
**HDL [mg/dL]**	**0.02**
**AST [U/L]**	**< 0.01**
**ALT [U/L]**	**< 0.01**
**GGT [U/L]**	**0.05**
Quick [%]	0.84
Thrombocytes [n/L]	0.99
Albumin [g/L]	0.61

**Table 4:** Correlation of MMP9 and indicated values with the respective correlation coefficient and *P*-value

Variable	Pearson’s r	*P*-value
Cholesterol [mg/dL]	-0.03	0.77
Triglycerides [mg/dL]	0.12	0.3
LDL [mg/dL]	-0.03	0.82
HDL [mg/dL]	-0.07	0.54
AST [U/L]	-0.07	0.52
ALT [U/L]	-0.01	0.91
GGT [U/L]	-0.05	0.69
BMI [kg/m²]	0.06	0.6
HbA1c [%]	0.19	0.1

## Discussion

NAFLD is one of the most prevalent liver diseases, especially in Western countries, making it one of the biggest health care challenges [3]. Despite its prevalence, noninvasive tests are currently lacking, while the present gold standard, liver biopsy, is rarely performed [6]. Based on the study of Coilly et al., MMP9 and FABP4 levels appeared to be promising candidates as serum markers for NAFLD [8]. The biobank offered the unique opportunity to simultaneously assess tissues, as well as serum markers. Histological examinations of liver tissue were performed to accurately diagnose NAFL/NASH and ELISAs for MMP9 and FABP4 levels in serum samples. Significantly elevated circulating MMP9 levels in patients with NASH based on the NAS were detected. In agreement with this, the NAS and MMP9 levels showed a positive correlation. Higher fibrosis scores were not associated with higher MMP9 levels.

### Comparisons with other studies and what does the current work add to the existing knowledge

Previously, two reports showed conflicting data for MMP9 levels in NAFLD-related liver fibrosis. Goyale et al. reported a decrease in circulating MMP9 levels in patients with more advanced fibrosis; however, this report lacked liver histology; therefore, it provides little evidence of MMP9 levels in patients with NASH [16]. Yilmaz et al. and Eren et al. did not observe a correlation between MMP9 levels and fibrosis scores [17]. In agreement with the later report, this study also did not find an association between MMP9 levels and fibrosis scores. However, only five patients had fibrosis scores of three or four. Therefore, the evidence remains limited for more advanced fibrosis stages.

In line with the present data, Yilmaz and Eren reported increased MMP9 levels in patients with NAFLD. They also performed liver biopsies but were likewise unable to distinguish patients with NAFL and NASH based solely on circulating MMP9 levels [17]. Nevertheless, data of this study provide evidence that MMP9 levels remain an attractive biomarker in NAFLD/NASH and that its combination with other markers is promising.

Elevated serum FABP4 levels in patients with obesity were previously reported [23]. However, data on circulating FABP4 levels in patients with NASH are scarce. Suh and colleagues reported increased serum FABP4 levels in patients with NAFLD. However, the included patients had normal weight or were overweight. Additionally, they did not perform liver biopsy [18]. Similar findings were reported recently, but this study also did not include liver biopsy [24]. This study shows that serum FABP4 levels were not increased in patients with NASH or fibrosis. It also did not detect a correlation between FABP4 levels and the NAS. Several possible reasons exist for this lack of correlation. One factor could be the source of FABP4. FABP4 is mainly expressed within adipose tissue. In obesity, FABP4 expression increases in the liver and decreases in the adipose tissue [23]. However, the increased expression in the liver might not contribute enough to the circulating levels of FABP4. Therefore, FABP4 is not the ideal candidate for noninvasive NASH diagnosis in patients with obesity.

This study was the first study, which investigated both MMP9 and FABP4 as markers for NASH. Furthermore, it investigated whether other routinely measured clinical parameters might improve the value of MMP9 to predict NAS. Hence, other clinical parameters were correlated with the NAS and chose the most promising candidates. Several markers associated with NAS, such as ALT, AST, HbA1c and MMP9 were identified. The developed models reached a fair discrimination with good AUC. Among all the different methods, the neural network fit best with the dataset, with an AUC of 83% and an accuracy of 88%. The AUC and accuracy of the neural network model showed that this test has the potential to be the starting point for future research. Other promising candidates to include in future analysis are cytokeratin-18 fragments (CK18) and soluble Fas [25]. Both are markers of apoptosis and could be a useful addition to MMP9, which is a marker of tissue remodeling [26].

### Strengths and limitations

The present study has several strengths and is the first to investigate the combination of circulating MMP9 and FABP4 concentrations as noninvasive tests for NASH. The strengths of this study include the paired biopsy and serum samples, as well as the robust and innovative model development.

Nevertheless, this study suffers from some limitations. The number of patients was relatively small, with only 84 patients included. However, even with a larger number of patients, it is not expected to find differing results. Additionally, an external validation of our findings is needed. Since patients with diabetes had higher MMP9 levels and higher NAS values, this study could not safely rule out that presence of diabetes influences MMP9 concentrations. However, MMP9 levels and HbA1c concentrations did not correlate with each other, therefore it is unlikely that diabetes severity influences MMP9 concentrations. Furthermore, all patients in this study underwent bariatric surgery. Hence, it is only possible to draw conclusions for patients with obesity.

## Conclusion

In summary, it was observed that serum MMP9 levels were associated with the NAS, while FABP4 serum levels were not. Additionally, MMP9 is a marker of tissue remodeling. Therefore, it should be routinely measured in patients with obesity before and after bariatric surgery. This should help to detect the course of liver pathology in these patients. Models were developed to predict NASH that included MMP9 as a predictor and achieved fair discrimination for patients with NASH. Since the test was not excellent, further investigations for a noninvasive NASH test are needed. Nevertheless, circulating MMP9 levels serve as a good candidate to be included in the clinical routine and future research.

## Data Availability

The datasets used and/or analysed during the current study are available from the corresponding author on reasonable request.

## Abbreviations

ALT: Alanine transaminase
ANOVA: Analysis of variance
AST: Aspartate transaminase
AUC: Area under the curve
BMI: Body mass index
CK18: Cytokeratin-18
DT: Decision tree
ELISA: Enzyme-linked Immunosorbent Assay
FABP4: Fatty acid-binding protein-4
GGT: Gamma-glutamyltransferase
HDL: High-density lipoprotein
MMP9: Matrix metalloproteinase-9
NAFL: Nonalcoholic fatty liver
NAFLD: Nonalcoholic fatty liver disease
NAS: NAFLD activity score
NASH: Nonalcoholic steatohepatitis
NN: Neural network
RF: Random Forest
ROC: Receiver operating characteristic
SD: Standard deviation
SPSS: Statistical package for social sciences
SVM: Support vector machine
T2D: Type 2 diabetes
